# Determination of c-myc amplification and overexpression in breast cancer patients: evaluation of its prognostic value against c-erbB-2, cathepsin-D and clinicopathological characteristics using univariate and multivariate analysis

**DOI:** 10.1038/sj.bjc.6693404

**Published:** 1999-12

**Authors:** A Scorilas, T Trangas, J Yotis, C Pateras, M Talieri

**Affiliations:** Departments of Virology and Biochemistry, ‘G Papanikolaou’ Research Center of Oncology, Hormone Receptor Unit, Breast Cancer Clinic, ‘St Savas’Hospital, Athens 11522, Greece

**Keywords:** c-myc, c-erbB-2, cathepsin-D, early relapse, relapse-free survival, overall survival, locoregional recurrence, breast cancer

## Abstract

C-myc and c-erbB-2 amplification and/or overexpression as well as total cathepsin-D (CD) concentration have been reported to be associated with poor prognosis in breast cancer. The prognostic significance, however, remains somewhat controversial, partly because of discrepancies among the different methodologies used. We determined the amplification and overexpression of c-myc oncogene in 152 breast cancer patients and examined its prognostic value in relation to c-erbB-2 amplification and overexpression, high concentration of CD (≥ 60 pmol mg^–1^ protein) and standard clinicopathological prognostic factors of the disease. High CD concentration, as well as c-myc amplification and overexpression, proved to be the best of the new variables examined for prediction of early relapse (ER; before 3 years). After multivariate analysis only CD remained significant, which suggests that the prognostic power of these variables is similar. Using univariate analysis we proved that c-myc amplification and overexpression were highly significant for disease-free survival (DFS) (*P* = 0.0016 and *P* = 0.0001 respectively) and overall survival (OS) (*P* < 0.0001 and *P* = 0.0095 respectively), although by multivariate analysis c-myc overexpression was statistically significant only for DFS (*P* = 0.0001) and c-myc amplification only for OS (*P* = 0.0006). With regard to c-erbB-2, only its overexpression appeared to be significant for DFS and OS, although after multivariate analysis its prognostic power was weaker (*P* = 0.030 and *P* = 0.024 respectively). c-myc amplification and overexpression exhibited a tendency for locoregional recurrence (LRR) (*P* = 0.0024 and *P* = 0.0075 respectively), however, their prognostic value was lower after multivariate analysis and only CD remained significant. © 1999 Cancer Research Campaign


*British Journal of Cancer* (1999) **81**(8), 1385–1391
© 1999 Cancer Research Campaign
Article no. bjoc.1999.0856

### Determination of c-myc amplification and overexpression in breast cancer patients: evaluation of its prognostic value against c-erbB-2, cathepsin-D and clinicopathological characteristics using univariate and multivariate analysis

**A Scorilas[1]*, T Trangas[1], J Yotis[2], C Pateras[3] and M Talieri[1]**

[1]Departments of Virology and Biochemistry, 'G Papanikolaou' Research Center of Oncology, [2]Hormone Receptor Unit, [3]Breast Cancer Clinic, 'St Savas' Hospital, Athens 11522, Greece

**Summary** C-myc and c-erbB-2 amplification and/or overexpression as well as total cathepsin-D (CD) concentration have been reported to be associated with poor prognosis in breast cancer. The prognostic significance, however, remains somewhat controversial, partly because of discrepancies among the different methodologies used. We determined the amplification and overexpression of c-myc oncogene in 152 breast cancer patients and examined its prognostic value in relation to c-erbB-2 amplification and overexpression, high concentration of CD ($\geq$ 60 pmol $mg^{-1}$ protein) and standard clinicopathological prognostic factors of the disease. High CD concentration, as well as c-myc amplification and overexpression, proved to be the best of the new variables examined for prediction of early relapse (ER; before 3 years). After multivariate analysis only CD remained significant, which suggests that the prognostic power of these variables is similar. Using univariate analysis we proved that c-myc amplification and overexpression were highly significant for disease-free survival (DFS) ($P = 0.0016$ and $P = 0.0001$ respectively) and overall survival (OS) ($P < 0.0001$ and $P = 0.0095$ respectively), although by multivariate analysis c-myc overexpression was statistically significant only for DFS ($P = 0.0001$) and c-myc amplification only for OS ($P = 0.0006$). With regard to c-erbB-2, only its overexpression appeared to be significant for DFS and OS, although after multivariate analysis its prognostic power was weaker ($P = 0.030$ and $P = 0.024$ respectively). c-myc amplification and overexpression exhibited a tendency for locoregional recurrence (LRR) ($P = 0.0024$ and $P = 0.0075$ respectively), however, their prognostic value was lower after multivariate analysis and only CD remained significant. © 1999 Cancer Research Campaign

**Keywords:** c-myc; c-erbB-2; cathepsin-D; early relapse; relapse-free survival; overall survival; locoregional recurrence; breast cancer

Despite major advances in therapy, survival of patients with breast cancer has not substantially improved. The search for reliable and sensitive prognostic tests is critical as they may help to identify patients for whom intensive adjuvant therapy is worthwhile. Histology alone is subjective and often not predictive of clinical behaviour. Many different tumour characteristics and cell components have been evaluated for prognostic significance in breast cancer. Regulatory and structural alterations of oncogenes appear to be one of the key events in the formation of most human cancers. Proto-oncogenes are present in all mammalian cells and are involved in normal growth and differentiation. Deregulated activation of the same genes can contribute to cancer development (Alitalo and Schwab, 1986). In particular, the presence of amplified oncogenes has been widely reported in human tumours, and in many cases a correlation of amplification degree with clinical indicators was detected.

*Received 15 December 1998*
*Revised 12 April 1999*
*Accepted 25 May 1999*

*Correspondence to:* M Talieri, Papanikolaou Research Centre of Oncology – Saint Savas Hospital, 171 Alexandras Avenue, Athens 11522, Greece

Expression of the c-myc proto-oncogene is involved in the regulation of cellular proliferation and terminal differentiation of human cells. Amplification of *c-myc* seems to associate with poor prognosis in breast cancer (Varley et al, 1987; Berns et al, 1992, 1996; Borg et al, 1992; Rous-Dosseto et al, 1992; Kreipe et al, 1993; Pertschuk et al, 1993; Pietilainen et al, 1995; Nass and Dickson, 1997) and to be a prognostic marker in node-negative patients (Berns et al, 1992; Borg et al, 1992). The *c-myc* oncogene produces a nuclear DNA-binding protein whose expression is regulated by oestrogen and down-regulated by tamoxifen in hormone-responsive human breast cancer in vitro (Santos et al, 1988; Van der Burg et al, 1989). Oestrogens are potent mitogens in a number of target tissues, including mammary glands where they play a pivotal role in the development and progression to mammary carcinoma. Oestrogens regulate the expression and function of c-myc and cyclin D1 and activate cyclin E–CdK2 complexes, all of which are rate-limiting for progression from G1 to S phase (Prall et al, 1998).

Reverse effects have been shown with respect to *c-erbB-2* expression (Dati et al, 1990; Read et al, 1990), i.e. oestrogen

*Present address: Department of Pathology and Laboratory Medicine, Mount Sinai Hospital and Department of Laboratory Medicine and Pathobiology, University of Toronto, 600 University Avenue, Toronto, Ontario M5G 1X5 Canada down-regulated the expression of c-erbB-2 and this effect was reversed by anti-oestrogens. It has been hypothesized that the activation of the two genes might share the same metabolic pathway; in fact, the c-erbB-2 gene has been identified as a cellular target for negative regulation by *c-myc* (Suen and Hung, 1991), whereas c-erbB-2 tyrosine kinase-mediated signals seem to down-regulate the immediate-early genes (Sistonen et al, 1990).

To express its full potential in systemic disease, however, the gene may have to act in concert with other events which also render the cell capable of metastasizing. Tumours with activated c-erbB-2 show several characteristics of the aggressive phenotype and are implicated in early relapse and shortened overall survival (Allred et al, 1998; Sjogren et al, 1998). One of the molecular mechanisms involved in the process of metastasis may be over-production of proteases that degrade the basement membrane and the extracellular matrix (Rochefort, 1992). The most extensively studied protease in human breast cancer is cathepsin-D (CD). Several reports on the prognostic value of CD in breast cancer have revealed poor survival for patients with high CD levels (Spyratos et al, 1989; Scorilas et al, 1993, 1995, 1999; Ferradina et al, 1997; Losch et al, 1998).

The present analysis was designed to extend and complete our previous work (Scorilas et al, 1993, 1995, 1999) and to assess the prognostic significance of the overexpression and amplification of *c-myc* in relation to various established prognostic factors as well as c-erbB-2 oncogene amplification and overexpression and CD concentration in Greek breast cancer patients, in an effort to better characterize this marker. The interrelationship was tested by univariate and multivariate analysis in a series of 152 breast cancer patients with a median follow-up of 5 years in our hospital.

### MATERIALS AND METHODS

### Patients

Tumour specimens from 152 patients (age: mean, 60 years; range 24–92 years) with no signs of distant metastasis and who underwent surgery for primary breast cancer from 1990 to 1995 (modified mastectomy 50 patients (32.9%); breast-conserving lumpectomy 102 patients (67.1%)) at the Oncologic Hospital of Athens ‘St Savas’, were evaluated in this study. Tumour specimens were drawn from a pool of frozen specimens originally submitted to the Laboratory of Hormone Receptors for steroid receptor analysis. Most of the women with positive lymph nodes generally received adjuvant chemotherapy (cyclophosphamide–methotrexate–5-fluorouracil for 6 cycles every 28 days; 70 patients); 102 patients received adjuvant (Tamoxifen) therapy (20 mg daily for 5 years), whereas 115 were irradiated. Twenty-one patients (13.8%) developed locoregional recurrence. Median follow-up for patients was 5 years (range 4–8 years). A computerized database containing updated information concerning each patient, together with receptor status, nodal status, size of the primary tumour, number of positive nodes, age and menopausal status of the patients, and/or differentiation grade of the tumour, was available for statistical analysis.

### Tumour sample processing

Tumour tissue was stored in liquid nitrogen. Samples were processed as we described previously (Scorilas et al, 1993, 1995). Tissue was pulverized in the frozen state and homogenized in 5 ml cytosol buffer (10 mM Tris, 1.5 mM EDTA, 5 mM NaMolybdate pH = 7.4, 5 mM dithiothreiol DTT). The homogenates were subjected to centrifugation at 40 000 rpm for 1 h at 4°C and the cytosols were kept at –80°C for later processing. The same cytosols were used for hormone receptors and for CD assays. DNA was isolated from 100 mg of tumour tissue, which was minced finely using a pair of scalpels, dispersed in 1 ml of 2 × TNE (20 mM Tris pH = 8.0, 300 mM NaCI, 20 mM EDTA) containing 0.5% sodium dodecyl sulphate (SDS) and digested with proteinase K (100 μg $ml^{-1}$) at 37°C. After repeated phenol, phenol–chloroform and chloroform–isoamyl alcohol extractions, intact genomic DNA was pooled following precipitation with 2 volumes of ethanol. RNA was isolated from frozen samples, ground to a fine powder in liquid nitrogen and subsequently homogenized in an acid guanidine thiocyanate–phenol–chloroform solution according to Chomczynski et al (1987). Southern blotting of *Eco*R1-digested DNA was performed by standard techniques (Feinberg and Vogelstein, 1983; Thomas, 1980). The integrity of the RNA was confirmed by formaldehyde-agarose gel electrophoresis. Northern blotting was performed according to Thomas (1980). Equal amounts of DNA (20 μg) were slot blotted on nylon membranes (Hybond $N^+$, Amersham). RNA (20 μg) was slot blotted according to Maniatis et al (1982).

### Detection of oncogenes

To determine c-myc overexpression or amplification, blots were hybridized overnight at 42°C to randomly primed [$\alpha$-$^{32}$P]dCTP-labelled c-myc probe (1.3 kb fragment, *Cla*I-*Eco*RI, from pHSR-1 plasmid-PBR 322-*Hin*dIII *Eco*RI-human genomic c-myc exon 3 from Colo 320-ATCC). To determine c-erbB-2 overexpression or amplification, blots were hybridized to c-erbB-2 (Oncogene Science) by a 5′ end labelling procedure (Promega), using [$\gamma^{32}$P]ATP. The hybridization was performed according to the instructions of the manufacturer and others (Miyada and Wallace, 1987). Briefly, after washing the blots at high stringency (2 × CCS, 0.1% SDS), autoradiography with intensifying screens was performed for 2–4 days at –70°C using Kodak X-OMAT-100 films and autoradiograms were scanned with a BioRad video densitometer 620. DNA and RNA extracted from paired normal breast tissue (obtained from radical mastectomies from areas distant to the cancer) was used as normal control. The values obtained for c-myc and c-erbB-2 by densitometer scanning were normalized to values obtained for *β-actin*. The ratios obtained were compared to average values obtained from 25 normal samples processed in order to determine amplification or overexpression.

### Hormone receptors

Oestrogen and progesterone receptors were assayed by ligand binding assay procedure using the dextran-coated charcoal technique as previously described (EORTC Breast Cancer Group, 1980; Kute et al, 1980). Results were expressed as specific binding sites per mg of cytosolic protein (fmol $mg^{-1}$ protein). The cut-off value for both oestrogen receptors and progesterone receptors was 10 fmol $mg^{-1}$ protein as established in our laboratory.

### Cathepsin-D assay

Total CD concentrations were measured using a standard assay (IRMA, ELISA Cath-D kit; CIS Bio International, Gif-sur-Yvette,

 © 1999 Cancer Research Campaign

**Table 1** Distribution of 152 patients on the basis of factors examined

| Factor | No. of patients | % |
|---|---|---|
| Age | | |
| ≤50 | 38 | 25.0 |
| 50–56 | 58 | 38.2 |
| >60 | 56 | 36.8 |
| Menopausal status | | |
| Pre/peri | 43 | 28.3 |
| Post | 109 | 71.7 |
| Tumour size | | |
| T1 | 44 | 29.0 |
| T2 | 81 | 53.3 |
| T3 | 27 | 27.7 |
| Lymph node status | | |
| Positive | 88 | 57.9 |
| Negative | 64 | 42.1 |
| Grade | | |
| I | 22 | 14.5 |
| II | 97 | 63.8 |
| III | 33 | 21.7 |
| Oestrogen receptor | | |
| Positive | 125 | 82.5 |
| Negative | 27 | 17.8 |
| Progesterone receptor | | |
| Positive | 130 | 85.5 |
| Negative | 22 | 14.5 |
| c-erbB-2 | | |
| Amplification | 31 | 20.4 |
| Overexpression | 45 | 29.6 |
| Cathepsin-D | | |
| Positive | 72 | 47.4 |
| Negative | 80 | 52.6 |
| C-myc | | |
| Overexpression | 43 | 28.3 |
| Amplification | 41 | 26.9 |
| 3 years relapse-free survival | 106 | 69.7 |
| Relapse-free survival[a] | 85 | 55.9 |
| Overall survival[a] | 104 | 68.4 |
| Locoregional recurrence | 21 | 13.8 |

[a]Median follow up 5 years

**Table 2** Univariate and multivariate analysis[a] for early recurrence of 152 primary breast cancer patients

| Factor | Univariate *P*-value | Multivariate *P*-value | Relative risk | 95% confidence intervals |
|---|---|---|---|---|
| C-myc amplification | 0.012 | NS | – | – |
| c-myc overexpression | 0.0088 | NS | – | – |
| Cathepsin-D | <0.0001 | 0.0001 | 3.12 | 2.32–4.19 |
| c-erbB-2 amplification | NS | NS | – | – |
| c-erbB-2 overexpression | NS | NS | – | – |
| Oestrogen receptor | 0.018 | NS | – | – |
| Progesterone receptor | NS | NS | – | – |
| Differentiation grade | 0.0084 | 0.035 | – | 1.07–3.20 |
| Lymph node status | 0.0018 | 0.0012 | 2.15 | 1.32–3.51 |
| Tumour size | 0.0027 | NS | – | – |
| Menopausal status | NS | NS | – | – |
| Age | 0.046 | NS | – | – |

[a]Cox regression analysis; NS, not significant ($P < 0.05$); median follow-up 60 months.

**Table 3** Univariate and multivariate analysis[a] for relapse-free survival of 152 primary breast cancer patients

| Factor | Univariate *P*-value | Multivariate *P*-value | Relative risk | 95% confidence intervals |
|---|---|---|---|---|
| c-myc amplification | 0.0016 | NS | – | – |
| c-myc overexpression | <0.0001 | 0.0001 | 2.25 | 1.55–3.05 |
| Cathepsin-D | 0.0022 | NS | – | – |
| c-erbB-2 amplification | NS | NS | – | – |
| c-erbB-2 overexpression | 0.031 | 0.0302 | 1.88 | 1.15–3.07 |
| Oestrogen recepter | 0.019 | 0.0030 | 0.25 | 0.11–0.63 |
| Progesterone recepter | 0.028 | NS | – | – |
| Differentiation grade | 0.0074 | NS | – | – |
| Lymph node status | 0.0055 | 0.0047 | 2.54 | 1.65–3.91 |
| Tumour size | 0.0010 | NS | – | – |
| Menopausal status | NS | NS | – | – |
| Age NS | | NS | – | – |

[a]Cox regression analysis; NS, not significant ($P < 0.05$); median follow-up 60 months.

France) according to the procedure described by the manufacturer in 1/40 and 1/80 dilutions of the reconstituted cytosols, both in duplicate.

### Statistics

Survival analyses were performed by constructing Kaplan–Meier DFS, OS and LRR curves (Kaplan and Meier, 1957), where differences between curves were evaluated by the log-rank test. Cox and logistic regression analysis were used to estimate the relative risks for relapse, locoregional recurrent and death (Tormod and Egil, 1985). Selection of prognostic variables with the highest significant effect in DFS, LLR and OS was performed in the Cox's model using the step-wise regression method in multivariate analysis. Only variables for which $P < 0.05$ were retained in the final model. Relative risks and 95% confidence intervals are presented only for retained variables, significant in the multivariate analysis. Tumour size and differentiation grade are continuous variables with scores 1–3.

### RESULTS

The frequency of amplification measured in 152 primary breast tumours was 26.9% for c-myc and 20.4% for c-erbB-2, whereas 47.4% of tumours produced high concentrations of CD (≥ 60 pmol $mg^{-1}$ protein). Moreover, overexpression of c-myc was found in 28.3% and of c-erbB-2 in 29.6% of tumours (Table 1). The magnitude of c-myc amplification ranged between 3 and 7 gene copies, whereas c-erbB-2 amplification ranged between 3 and 10 copies. The overexpression was stronger for c-erbB-2 (3–12 times) than for c-myc (3–6 times) (data not shown). The median CD concentration was 59 pmol $mg^{-1}$ protein (range 23.2–132 pmol $mg^{-1}$ protein). The patients examined were divided in subgroups: (1) according to survival: early relapse (ER; before 3 years); disease-free survival (DFS; median 5 years); overall survival (OS; median 5 years) and (2) according to locoregional recurrence (LRR).

### Statistic analysis for the ER

By univariate analysis, the variables found to negatively affect ER were CD, c-myc amplification, lymph node status, tumour size, differentiation grade, c-myc overexpression and age. ER was

© 1999 Cancer Research Campaign

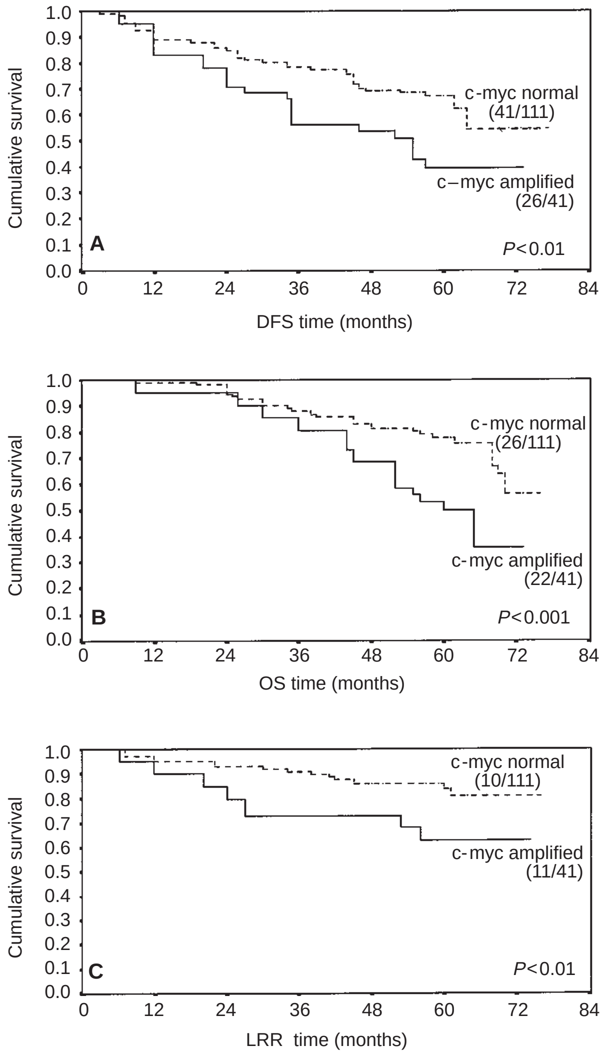

**Figure 1** Kaplan–Meier survival plots of DFS (**A**), OS (**B**) and LRR (**C**) of 152 patients with c-myc normal and c-myc amplified. Differences among the two groups for DFS, OS and LRR were determined by log-rank test. Numbers in parentheses indicate the number of failures/total number of patients in each group

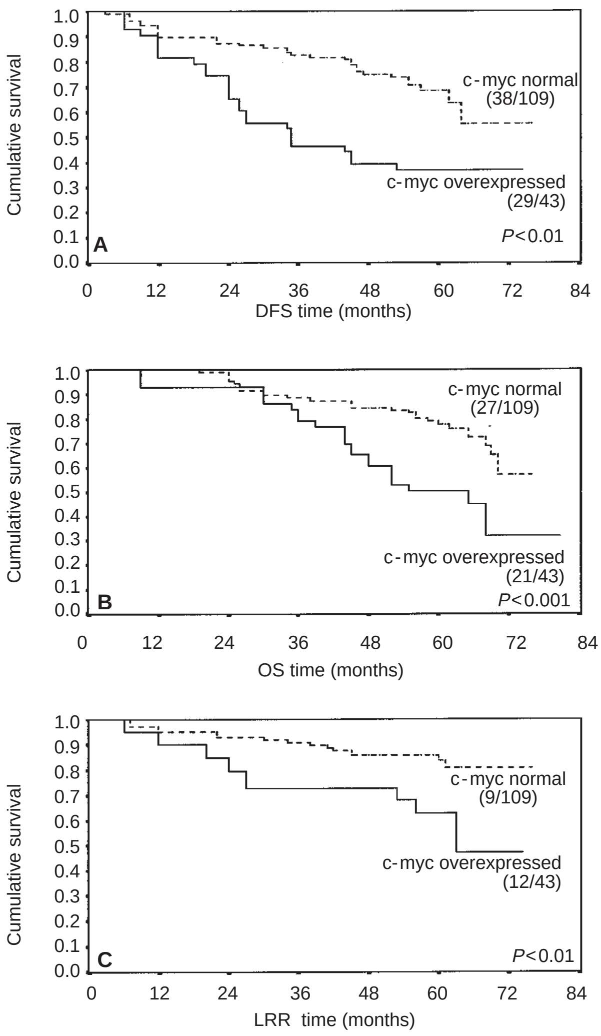

**Figure 2** Kaplan–Meier survival plots of DFS (**A**), OS (**B**) and LRR (**C**) of 152 patients with c-myc normal and c-myc overexpressed. Differences among the two groups for DFS, OS and LRR were determined by log-rank test. Numbers in parentheses indicate the number of failures/total number of patients in each group positively affected by oestrogen receptor but was unaffected by menopausal status, progesterone receptor, or amplification and overexpression of c-erbB-2. Multivariate analysis again reveals CD ($P$ = 0.0001) as the most important variable influencing ER and the precision of the prediction is statistically improved when lymph node status and grade ($P$ = 0.012 and $P$ = 0.035 respectively) are considered (Table 2). In a previous study we showed that the concentration of CD was found to be positively correlated with c-myc amplification and overexpression (Scorilas et al, 1993). This finding, by multivariate analysis, reduces the significance of c-myc oncogene, due to technically easier determination of CD.

### Univariate and multivariate analysis for DFS

Relapse free survival as shown in Table 3 was negatively affected by c-myc overexpression and amplification, tumour size, CD concentration, lymph node status, differentiation grade and c-erbB-2 overexpression; however, it was positively affected by the oestrogen and progesterone receptors, and remained unaffected by age and c-erbB-2 amplification. By multivariate analysis, c-myc overexpression and lymph node status emerged as the variables with the strongest influence on DFS. Prediction, however, is improved statistically when oestrogen receptor and C-erbB-2 overexpression are considered. In our previous study (Scorilas et al, 1993) we reported positive correlation between c-myc overexpression and amplification. This reduces the significance of c-myc amplification as shown by multivariate analysis. The Kaplan–Meier curves (Figures 1A and 2A) also show that patients with c-myc overexpression or amplification have a smaller probability for longer DFS than patients without either of them. The difference in DFS effect was greater for c-myc overexpression than for c-myc amplification.

### Statistic analysis for OS

Overall survival is negatively influenced by the following variables: c-myc amplification and overexpression, tumour size,

*© 1999 Cancer Research Campaign*

**Table 4** Cox univariate and multivariate analysis[a] for the overall survival of 152 primary breast cancer patients

| Factor | Univariate *P*-value | Multivariate *P*-value | Relative risk | 95% confidence intervals |
|---|---|---|---|---|
| c-myc amplification | <0.0001 | 0.0006 | 3.10 | 2.18–4.41 |
| c-myc overexpression | 0.0095 | NS | – | – |
| Cathepsin-D | NS | NS | – | – |
| c-erbB-2 amplification | NS | NS | – | – |
| c-erbB-2 overexpression | 0.0021 | 0.024 | 1.62 | 1.01–2.59 |
| Oestrogen receptor | 0.0006 | 0.0053 | 0.48 | 0.32–0.71 |
| Progesterone receptor | 0.0041 | 0.025 | 0.52 | 0.29–0.94 |
| Differentiation grade | 0.042 | NS | – | – |
| Lymph node status | 0.0081 | 0.0043 | 3.30 | 2.18–4.98 |
| Tumour size | 0.0021 | 0.015 | 1.84 | 1.15–2.94 |
| Menopausal status | NS | NS | – | – |
| Age | NS | NS | – | – |

[a]Cox regression analysis; NS, not significant ($P < 0.05$); median follow-up 60 months.

**Table 5** Univariate and multivariate analysis[a] for locoregional recurrence of 152 primary breast cancer patients

| Factor | Univariate *P*-value | Multivariate *P*-value | Relative risk | 95% confidence intervals |
|---|---|---|---|---|
| c-myc amplification | 0.0024 | NS | – | – |
| c-myc overexpression | 0.0075 | NS | – | – |
| Cathepsin-D | 0.0016 | 0.0067 | 4.2 | 2.52–6.99 |
| c-erbB-2 amplification | 0.0022 | 0.0091 | 2.7 | 1.50–4.86 |
| c-erbB-2 overexpression | NS | NS | – | – |
| EsR | 0.0063 | 0.019 | 0.32 | 0.20–0.52 |
| PgR | 0.012 | NS | – | – |
| Differentiation grade | NS | NS | – | – |
| Lymph node status | NS | NS | – | – |
| Tumour size | 0.018 | NS | – | – |
| Menopausal status | NS | NS | – | – |
| Age | NS | NS | – | – |

[a]Cox regression analysis; NS, not significant ($P < 0.05$); median follow-up 60 months.

c-erbB-2 overexpression, lymph node status and differentiation grade (Table 4). Again, the role of oestrogen and progesterone receptors is protective, whereas age, menopausal status, CD and c-erbB-2 amplification do not seem to influence OS. The Kaplan–Meier survival curves (Figures 1B and 2B) show the reduced probability of patients with c-myc amplification or c-myc overexpression for OS in contrast to those without the two markers. Using multivariate analysis we proved that OS can be predicted by combining the variables: c-myc amplification, lymph node involvement and oestrogen receptor. The prediction is improved if progesterone receptor, tumour size and c-erbB-2 overexpression are taken into account as well.

### Analysis for LRR

We observed that 27% and 28% of patients with c-myc amplification and overexpression respectively developed locoregional recurrence, while only 9% and 8% of patients without c-myc amplification and overexpression respectively had locoregional recurrence (Figures 1C and 2C). In Cox univariate analysis, c-myc amplification, c-myc overexpression, CD concentration, c-erbB-2 amplification and tumour size have a positive effect on locoregional recurrence, while oestrogen and progesterone receptors have a negative effect (Table 5). The Kaplan–Meier curves (Figures 1C and 2C) also show that patients with c-myc amplification or overexpression have a greater chance of developing LRR than patients without them. Multivariate analysis suggests that high CD concentration is the most important variable for LRR. The positive correlation between CD concentration and c-myc amplification and overexpression, reported previously by our group (Scorilas et al, 1993), reduces in multivariate analysis the role for c-myc determination for prediction of disease course.

### DISCUSSION

Breast cancer is the most common form of malignancy among women today. It would be beneficial for patients to have tools available that could more reliably predict the rate of recurrence in primary breast cancer, in addition to the classical prognostic factors. In recent years, many biological markers have been studied for their correlation with prognosis (McGuire et al, 1991; Gasparini et al, 1992; Osborne et al, 1992; Foekens et al, 1996; Nass and Dickinson, 1996; Thor and Yaudell, 1996). The myc family of nuclear proto-oncogenes plays critical roles during cell growth, differentiation and transformation. Although the molecular mechanisms underlining myc-mediated cellular transformation are still under investigation, evidence is accumulating that c-myc transcriptionally controls the expression of a diverse group of genes, and that its deregulation leads to a cellular imbalance in the expression of genes that control both proliferation and death.

Amplification of the c-myc locus in breast cancer tissue has been observed in many studies (Berns et al, 1992*a*, 1992*b*; Borg et al, 1992; Watson et al, 1993; Lonn et al, 1995). The reported frequency of amplification varies greatly (from 4% to 52%) in these studies, but the overall mean appears to be about 20%. There is also considerable variability in the predictive value of c-myc amplification and correlation with other prognostic markers of breast cancer. Some reports indicate that c-myc amplification is predictive for shortened relapse-free and/or overall survival (Berns et al, 1992*a*; Borg et al, 1992; Lonn et al, 1995), while Berns et al (1995) upon concurrent examination of c-erbB-2, c-myc and int-2 showed that c-myc was the only oncogene whose amplification was significantly related with the rate of relapse.

In addition, a number of studies have examined c-myc expression in breast cancer at both the mRNA and protein levels. Northern analysis indicated that c-myc mRNA expression was elevated compared to that observed in normal breast tissue in 70% (Escot et al, 1986) or 45% (Garcia et al, 1989) of breast tumours.

Immunohistochemistry also has been frequently used to examine the relative levels of myc protein in mammary tumour specimens (Pavelic et al, 1992; Pietilainen et al, 1995). Tulchin et al (1996), using immunohistochemistry, have shown continuity of c-myc expression during tumour progression, whereas Bland et al (1995), while studying the co-expression of c-myc with other oncogenes, report that co-expression of c-myc, Ha-ras and c-fos function as a strong prognostic correlate for recurrence and survival. Variation in results throughout the literature is not surprising given the broad range of sample size, composition and follow-up, as well as inconsistencies in experimental and statistical methodology.

© 1999 Cancer Research Campaign 

In the present study, the distribution of c-myc amplification and overexpression, c-erbB-2 amplification and overexpression and high CD concentration (Table 1) are in agreement with data reported in the literature. With respect to the prognostic value of c-erbB-2 for DFS and OS, we observed a discriminative power only for its overexpression, which was reduced in multivariate analysis (Tables 3 and 4). More important is our finding concerning the clinical impact of c-myc amplification and overexpression. This study (Tables 3 and 4) suggests that both are highly significant for DFS and OS of patients, albeit in multivariate analysis only c-myc overexpression for DFS and c-myc amplification for OS remain statistically significant. For ER the best predictors of the new markers examined are high CD concentration, c-myc amplification and c-myc overexpression (Table 2), but following multivariate analysis, only the CD remains relevant, which suggests that the prognostic power of the three variables is not additive. In general, the prognostic relevance of c-myc amplification and overexpression overlap as we reported (Scorilas et al, 1993) and the determination of one of them only is sufficient. The association of LRR with high CD concentration, c-erbB-2 amplification and c-myc amplification and overexpression is also important. Nevertheless, their impact is not additive and following multivariate analysis only high CD concentration remains statistically significant and can be exploited as a marker for modification of patient treatment (Table 5). The present study also reveals that for ER and LRR, c-erbB-2 amplification and overexpression have smaller prognostic value from the c-myc amplification and overexpression, although for the former these values are additive (Table 2 and 5). For ER the only new variable which has significance in multivariate analysis is high CD (Table 2). The observation, which to the best of our knowledge has not been reported previously, is the positive association between c-myc and LRR. Therefore, we can propose that patients showing c-myc amplification or overexpression have a tendency for locoregional recurrence, which could be exploited as a marker for modification of patients' treatment.

In conclusion, c-myc amplification can be used as a prognosticator for overall survival, whereas c-myc overexpression for relapse-free survival of breast cancer patients. The best of the new predictors for early relapse remains high CD concentration, while c-myc amplification and overexpression manifest a tendency for locoregional recurrence.

### REFERENCES

Alarscon R, Rupnow B, Graeber T, Knox S and Giaccia A (1996) Modulation of c-myc activity and apoptosis in vivo. *Cancer Res* **56**: 4315–4319

Alitalo K and Schwab M (1986) Oncogene amplification in tumour cells. *Adv Cancer Res* **47**: 235–281

Allred DC, Harvey JM and Clark GM (1998) Prognostic and predictive factors in breast cancer by immunohistochemical analysis. *Mod Pathol* **11**: 155–168

Ben-Yosef T, Yanuka O, Halle D and Benvenistry N (1998) Involvement of myc targets in c-myc and N-myc induced human tumours. *Oncogene* **17**: 165–171

Berns EMJJ, Klijn JGM, van Puten WLJ, van Staveren IL, Portengen H and Foekens JA (1992*a*) c-myc amplification is a better prognostic factor than HER2/neu amplification in primary breast cancer. *Cancer Res* **52**: 1107–1113

Berns EMJJ, Klijin JGM, van Staveren IL, Portengen H, Noordegraaf E, Foekens JA (1992*b*) Prevalence of amplification of the oncogenes c-myc, HER2/neu, and int-2 in one thousand human breast tumours: Correlation with steroid receptors. *Eur J Cancer* **28**: 697–700

Berns EM, Klijin JG, Smid M, van Staveren IL, Look MP, van Putten WL and Foekens JA (1996) TP53 and MYC gene alterations independently predict poor prognosis in breast cancer patients. *Genes Chromosomes Cancer* **16**: 170–179

Bland KI, Konstadoulakis MM, Vezeridis MP and Wanebo HJ (1995) Oncogene protein co-expression: value of Ha-ras, c-myc, c-fos, and p53 as prognostic discriminants for breast carcinoma. *Ann Surg* **221**: 706–720

Chomczynski P and Sacchi N (1987) Single-step method of RNA isolation by acid guanidinium thiocyanate-phenol-chloroform extraction. *Anal Biochem* **162**: 156–159

Dati C, Antoniotti S, Taverna D, Perroteau I and De Bortoli M (1990) Inhibition of c-erbB-2 oncogene expression by estrogens in human breast cancer cells. *Oncogene* **5**: 1001–1006

Dubik D and Shiu RPC (1988) Transcriptional regulation of *c-myc* oncogene expression by oestrogen in hormone-responsive human breast cancer cells. *J Biol Chem* **263**: 12705–12708

Escot C, Theillet C, Lidereau R, Spyratos F, Champeme MH, Gest J and Callahan R (1986) Genetic alteration of the c-myc protooncogene (MYC) in human primary breast carcinomas. *Proc Natl Acad Sci USA* **83**: 4834–4838

Feinberg A and Vogelstein B (1984) A technique for labelling DNA restriction fragments to a high specific activity. *Anal Biochem* **137**: 66–67

Ferrandina G, Scambia G, Benedetti-Panici P, Mancuso S and Messori A (1997) Relationship between Cathepsin-D content and disease-free survival in node-negative breast cancer patients: a meta-analysis. *Br J Cancer* **76**(5): 661–666

Foekens JA, Berns EMJJ, Look MP et al (1996) Prognostic factors in node-negative breast cancer. In: *Hormone-Dependent Cancer,* Pasqualini JR and Katzenellebogen BS (eds), pp. 217–253. Dekker: New York

Garcia I, Dietrich PY, Aapro M, Vauthier G, Vadas L and Engel F (1989) Genetic alteration of *c-myc*, *c-erbB-2* and *c-Ha-ras* protooncogenes and clinical associations in human breast carcinomas. *Cancer Res* **49**: 6675–6679

Gasparini G, Gozza F and Harris AL (1992) Evaluating the potential usefulness of new prognostic and predictive indicators in axillary node-negative breast patients. *J Natl Cancer Inst* **12**: 1006–1014

Guerin M, Barrois M and Terrier M (1988) Overexpression of either *c-myc* or *c-erbB-2* (*neu*) proto-oncogene in human breast carcinomas: correlation with poor prognosis. *Oncogene Res* **3**: 21–31

Kaplan EL and Meier P (1958) Non-parametric estimation from incomplete observations. *J Am Stat Assoc* **53**: 457–481

Kreipe H, Feist H, Fischer L, Felgner J, Heidorn K, Mettler L and Parwaresch R (1993) Amplification of c-myc but not of c-erbB-2 is associated with high proliferative capacity in breast cancer. *Cancer Res* **53**: 1956–1961

Lonn U, Lonn S, Nilsson B and Stenkvist B (1995) Prognostic value of erb-B2 and myc amplification in breast cancer imprints. *Cancer* **75**: 2681–2687

Losch A, Templer C, Kohlberger P, Joura EA, Denk M, Zajic B, Breitenecker G and Kainz C (1998). Prognostic value of cathepsin-D expression and association with histomorphological subtypes in breast cancer. *Br J Cancer* **78**: 205–209

Maniatis T, Fritsch EF and Sambrock J (1982) *Molecular Cloning: a Laboratory* Manual. Cold Spring Harbour Press: New York

McGuire WL (1991) Breast cancer prognostic factors: evaluation guidelines. *J Natl Cancer Inst* **83**: 154–155

Miyada CG and Wallace RB (1987) Oligonucleotide hybridization techniques Methods in enzymol. **154**: 94–107

Nass SJ and Dickson RB (1997) Defining a role for c-myc in breast tumorigenesis. *Breast Cancer Res Treat* **44**: 1–22

Osborne CK (1992) Prognostic factors for breast cancer: have they met their promise? *J Clin Oncol* **10**: 679–682

Pavelic ZP, Pavelic L, Lower EE, Gapany S, Barker EA and Preisler HD (1992) c-myc, c-erbB-2 and Ki-67 expression in normal in breast tissue and in invasive and non invasive breast carcinoma. *Cancer Res* **52**: 2597–2602

Pertschuk LP, Feldman JG, Kim DS, Nayeri K, Eisenberg KB, Carte AC, Thelmo WT, Rhong ZT, Benn P and Grossman A (1993) Steroid hormone receptor immunohistochemistry and amplification of c-myc protooncogene. Relationship to disease-free survival in breast cancer. *Cancer* **71**: 162–171

Pietilainen T, Lipponen P, Aaltomaa S, Eskelinen M, Kosma VM and Syrjanen K (1995) Expression of c-myc proteins in breast cancer as related to established prognostic factors and survival. *Anticancer Res* **15**: 959–964

Prall OW, Rogan EM and Sutherland RL (1998) Estrogen regulation of cell cycle progression in breast cancer cells. *J Steroid Biochem Mol Biol*: **65**: 169–174

Read LD, Keith D Jr, Slamon DJ and Katzenellebogen BS (1990) Hormone modulation of HER2/neu protooncogene messenger ribonucleic acid and p185 protein expression in human breast cancer cell lines. *Cancer Res* **50**: 3947–3951

Rochefort H (1992) Biological and clinical significance of cathepsin-D in breast cancer. *Acta Oncol* **31**: 125–130

Roux-Dosseto M, Romain S, Dussault N, Desideri C, Piana L, Bonnier P et al (1992) c-myc gene amplification in selected node-negative breast cancer patients correlates with high rate of early relapse. *Eur J Cancer* **28A**: 1600–1604

© 1999 Cancer Research Campaign

Santos GF, Scott GK, Lee WMF, Liu E and Benz C (1988) Estrogen-induced posttranscriptional modulation of *c-myc* proto-oncogene expression in human breast cancer cells *J Biol Chem* **263**: 9565–9568

Scorilas A, Yotis J, Gouriotis D, Keramopoulos A, Ampela K, Trangas T and Talieri M (1993) Cathepsin-D and c-erbB-2 have an additive prognostic value for breast cancer patients. *Anticancer Res* **13**: 1895–1900

Scorilas A, Yotis J, Stravolemos K, Gouriotis D, Keramopoulos A, Ampela K, Talieri M and Trangas T (1995) *c-erbB-2* overexpression may be used as an independent prognostic factor for breast cancer patients. *Anticancer Res* **15**: 1543–1548

Scorilas A, Yotis J, Pateras Ch, Trangas T and Talieri M (1999) Predictive value of *c-erbB-2* and Cathepsin-D for Greek breast cancer patients using univariate and multivariate analysis. *Clinical Cancer Res* **5**: 815–821

Sistonen L, Koskinen PJ, Lehvaslaiho H, Lehtola L, Bravo R and Alitalo K (1990) Down regulation of the early genomic growth factor response in neu oncogene transformed cells. *Oncogene* **5**: 815–821

Sjogren S, Inganas M, Lindgren A, Holmberg L and Bergh J (1998) Prognostic and predictive value of c-erbB-2 overexpression in primary breast cancer, alone and in combination with other prognostic markers. *J Clin Oncol* **16**: 462–469

Spyratos F, Maudelonde T, Brouillet JP, Brunet M, De-Fene A, Andrieu C, Harene K, Desplaces A, Rouesse J and Rochefort H (1989) Cathepsin-D: an independent prognostic factor for metastasis of breast cancer. *Lancet* **ii**: 1115–1118

Suen TC and Hung MC (1991) *c-myc* reverses *neu*-induced transformed morphology by transcriptional repression. *Mol Cell Biol* **11**: 354–362

Thomas PS (1980) Hybridization of denatured RNA and small DNA fragments transferred to a high specific activity. *Proc Natl Acad Sci USA* **77**: 5201–5205

Thor AD and Yandell DW (1996) Molecular pathology of breast carcinoma. In: *Diseases of the Breast*, Harris JR, Lippman ME, Morrow M and Hellman S (eds), pp. 221–235. Lippincott-Raven: Philadelphia

Tormod B and Egil A (1985) Selecting factors: a comparison of discriminant analysis logistic regression and Cox's regression model using data from the Tromso heart study. *Stat Med* **4**: 413–423

Van der Burg B, van Selm-Miltenburg AJP, de Last SW and van Zoolen EJJ (1989) Direct effects of estrogen on *c-fos* and *c-myc* protooncogene expression and cellular proliferation in human breast cancer cells. *Mol Cell Endocrinol* **64**: 223–228

Watson PH, Singh R and Hole AK (1996) Influence of *c-myc* on the progression of human breast cancer. *Curr Top Microbiol Immunol* **213**: 267–283

*© 1999 Cancer Research Campaign*
